# Association Between Child Abuse Experience and Pathological Internet Use Among Chinese University Students: The Mediating Roles of Security and Maladaptive Cognitions

**DOI:** 10.3389/fpsyg.2022.830031

**Published:** 2022-04-07

**Authors:** Ningbo Qin, Pei Li, Yu Tian

**Affiliations:** ^1^Department of Marxism, Hebei University of Technology, Tianjin, China; ^2^Tsingtao Beer Museum, Qingdao, China; ^3^Department of Marxism, Qingdao University of Science and Technology, Qingdao, China

**Keywords:** pathological internet use, child abuse, security, maladaptive cognitions, mediating roles

## Abstract

Research has revealed that child abuse experience can increase pathological Internet use; however, few studies have focused on the influence of child abuse experience on pathological Internet use. This study examined the mediating roles of security and maladaptive cognitions in the association between child abuse and pathological Internet use. A total of 918 Chinese university students participated in the study, with measurements of child abuse, security, maladaptive cognitions, and pathological Internet use being employed. Structural equation modeling results indicated that child abuse could positively predict (i) pathological Internet use, (ii) pathological Internet use through the mediating role of security, (iii) pathological Internet use through the mediating role of maladaptive cognitions, and (iv) pathological Internet use through the chain mediating role of security and maladaptive cognitions. These results indicated that security and maladaptive cognitions were the primary factors in the association between child abuse and pathological Internet use.

## Introduction

With the increasing ubiquity of the Internet, an increasing number of people are exhibiting pathological Internet use, which is defined as excessive or compulsive Internet use and a preoccupation with and loss of control over Internet use, resulting in negative personal and professional consequences (Davis, 2001; Caplan, 2002; also known as problematic Internet use or Internet addiction) that are detrimental to these individuals (Greenfield and Yan, 2006). Studies have revealed that pathological Internet use is associated with numerous physical health problems (Kuss et al., 2021), psychological dysfunction (Robbins and Clark, 2015; Kuss and Lopez-Fernandez, 2016), and emotional disorders (Kitazawa et al., 2018; Hernández et al., 2019). Therefore, an increasing number of studies have examined the predictors of pathological Internet use.

Studies have indicated that child abuse, which encompasses physical abuse, sexual abuse, emotional neglect, physical neglect, and emotional abuse (Bernstein et al., 2003), is associated with many addictive behaviors (Siomos et al., 2012; Chen et al., 2017; Lo et al., 2021); however, the influences of pathological Internet use have received limited research attention. According to the social compensation model, individuals’ lack of personal offline relationships, especially among introverted individuals, may lead them to compensate through Internet use (Morgan and Cotton, 2004; Frison and Eggermont, 2016). Additionally, social compensation model also suggested that compensation can be divided into constructive compensation and pathological compensation. Constructive compensation refers to the transfer of individual needs by other activities, which can repair individuals’ needs and promote individuals’ physical and mental development, while pathological compensation cannot repair individuals’ needs and hinder individuals’ physical and mental development (Morgan and Cotton, 2004). Obviously, the constructive compensation could protect individuals to suffer pathological Internet use, while pathological compensation could increase the risk of pathological Internet use. However, numerous studies have indicated that individuals who were abused during their childhood were more likely to have personal relationship problems (Fernet et al., 2019; Yoon et al., 2021). Therefore, those individuals may use the Internet more often to overcome stress or social anxiety, potentially increasing the likelihood of pathological Internet use and developing into pathological compensation.

### Mediating Roles of Security and Maladaptive Cognitions

Studies have revealed that child abuse is negatively associated with perceived security, which was defined as the premonition of possible physical or psychological danger or risk, as well as the sense of strength and powerlessness of individuals in dealing with it, which is mainly manifested in the sense of certainty and control (Boisjoli et al., 2019; Tierolf et al., 2020). Because individuals who were abused during their childhood were more likely to exhibit personal relationship problems, which could increase their sense of rejection and level of insecurity (Hadlington and Parsons, 2017). However, security is a vital predictor of pathological Internet use because communicating, playing games, and browsing the news can increase individuals’ perceived security and sense of control, which may lead them to use the Internet more often (Hadlington and Parsons, 2017; Jia et al., 2017). Therefore, child abuse may increase individuals’ pathological Internet use through the mediating role of security.

Additionally, in identifying the predictors of pathological Internet use, the cognitive behavioral model offers theoretical explanations of the origins and pathogenesis of pathological Internet use. Davis (2001) suggested that psychopathology (such as depression and social anxiety) is a distal necessary cause of symptoms of pathological Internet use. Notably, the key factors of pathological Internet use are maladaptive cognitions, which are proximal sufficient causes, and distal causes that could influence pathological Internet use through proximal sufficient causes (Davis, 2001; Caplan, 2002). Maladaptive cognitions of the Internet is defined as distorted cognitions, such as “I feel that online life is more wonderful and exciting than real life,” which could lead individuals to spend more time on the Internet. From this point of view, maladaptive cognitions may play an important role in the association between child abuse and pathological Internet use. For example, individuals tend to deem the Internet a safe place to communicate with others because they can overcome social anxiety and apprehension when safely sitting behind a screen (Yao and Zhong, 2014; Tian et al., 2020). However, some studies have reported that child abuse was positively associated with social anxiety and apprehension (Robertson and Milner, 1985; Trent et al., 2021), which indicates that child abuse may increase individuals’ pathological Internet use through the mediating role of maladaptive cognitions. Additionally, security, which is significant related with social anxiety (Hadlington and Parsons, 2017), appears to be a distal cause of pathological Internet use for the significant association. Therefore, child abuse may also influence pathological Internet use through the sequential mediating role of security and maladaptive cognitions.

### University Students

Numerous studies have suggested that university students are more likely than other individuals to exhibit pathological Internet use because have they have more free time (Morahan-Martin and Schumacher, 2000; Chen, 2012); therefore, this population was investigated in the current study. Rather than using the designated time to study, some students spend their free time on the Internet, thus increasing the likelihood of pathological Internet use. Additionally, students’ access to smartphones, iPads, and computers provide them with more opportunities to browse the Internet and possibly develop pathological Internet use.

### The Present Study

**FIGURE 1:**
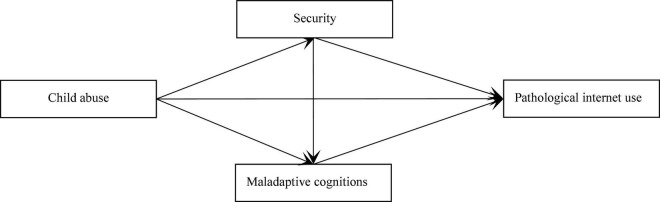
Proposed model.

According to the social compensation model, individuals may attempt to compensate for a lack of personal offline relationships through pathological Internet use. Because child abuse is, to some extent, defined as the absence of a childhood, not having a childhood may be a vital predictor of pathological Internet use. Therefore, this study examined the influence of child abuse on pathological Internet use. Additionally, on the basis of the cognitive behavioral model, we also examined the mediating roles of security and maladaptive cognitions in the association between child abuse and pathological Internet use. With reference to the literature, we proposed a dual-path mediation model (Figure 1) and proposed the following hypotheses:

1. Child abuse would positively predict pathological Internet use among university students.
2. Child abuse would positively predict pathological Internet use through the mediating role of security.
3. Child abuse would positively predict pathological Internet use through the mediating role of maladaptive cognitions.
4. Child abuse would positively predict pathological Internet use through the sequential mediating roles of security and maladaptive cognitions.

## Materials and Methods

### Participants

A total of 918 Chinese university students (395 men and 523 women; *M*_age_ = 19.34 years, *SD* = 0.76 years) from a university in northeast China participated in the survey with cluster sampling method. The study was conducted in November 2020. A research team consisting of a teacher and postgraduate students conducted the survey in three steps. First, students from several classes were recruited to participate in the research (participation was voluntary). Second, the researchers briefed the students on the purpose of the study, answered questions, and informed them that they could ask the researchers for help if necessary. Third, all participants received a small compensatory gift. And all the students signed the informed consent. The present study was conducted in accordance with the 1964 Helsinki declaration and its later amendments or comparable ethical standards, with the approval of the Human Research Ethics Committee of Qingdao University of Science and Technology.

### Measures

#### Child Abuse Scale

The Chinese version of the Child Abuse Scale was used to measure students’ experience of child abuse (Zhang, 2007). The Child Abuse Scale consists of 28 items assessed with a 5-point Likert scale, ranging from 1 (*never*) to 5 (*often*). Items included “Someone in my family hit me, leaving bruises or scars,” and “I think someone in my family hates me.” The total scores of the 28 items were deemed the indicator of the extent of each student’s child abuse, with higher scores representing greater abuse. The Cronbach’s α coefficient of this scale was 0.86.

#### Security Scale

The Chinese version of the Security Scale was used to measure students’ perceived level of security (Zhong, 2004). The Security Scale consists of 16 items assessed with a 5-point Likert scale, ranging from 1 (*completely inconsistent*) to 5 (*completely consistent*). Items included, “I always worry about what will happen,” and “People say I am a shy person.” The total scores of the 16 items were deemed the indicator of each student’s perceived sense of security, with higher scores representing lower security. The Cronbach’s α coefficient of the scale was 0.91.

#### Maladaptive Cognitions Scale

The Chinese version of the Maladaptive Cognitions Scale was used to measure the consistency of students’ maladaptive cognitions (Li et al., 2010). The Maladaptive Cognitions Scale comprises 14 items assessed with a 5-point Likert scale, ranging from 1 (*completely inconsistent*) to 5 (*completely consistent*). Items included, “I feel very safe online than real life,” and “I can be myself when I surf the Internet rather than real life.” The total scores of the 14 items were deemed the indicator of the extent of each student’s maladaptive cognitions, with higher scores representing more maladaptive cognitions. The Cronbach’s α coefficient of the scale was 0.91.

#### Pathological Internet Use Scale

The Chinese version of the Pathological Internet Use Scale was used to measure the level of pathological Internet use (Bai and Fan, 2005). The Pathological Internet Use Scale comprises 19 items assessed with a 5-point Likert scale, which ranges from 1 (*completely inconsistent*) to 5 (*completely consistent*). Items included, “I found myself betting on the Internet and reducing my interaction with my friends,” and “The internet has caused some negative effects on my study or work.” The total scores of the 19 items were deemed the indicator of each student’s pathological Internet use, with higher scores representing greater pathological Internet use. The Cronbach’s α coefficient of the scale was 0.95.

### Statistical Analyses

Statistical analyses consisted of two steps: (i) conducting descriptive statistical analysis, correlation analysis, and gender difference tests with SPSS 21.0, and (ii) examining the mediating roles of security and maladaptive cognitions in the association between child abuse and pathological Internet use through structural equation modeling (SEM), conducted with MPLUS 7.0. The total child abuse, security, maladaptive cognitions, and pathological Internet use scores were used as the observed variable in all statistical analyses. Additionally, the χ^2^ test, comparative fit index (CFI), Tucker–Lewis index (TLI), and root-mean-square error of approximation (RMSEA) were employed to assess the model fit. A TLI and CFI of >0.95 and RMSEA values of ≤0.08 indicated a suitable model fit (Browne and Cudeck, 1993). The χ^2^ test of difference (Δχ^2^) was used to compare the fit of the nested models.

## Results

### Descriptive Statistics, Correlation Analysis, and Gender Differences

The means and standard deviations for child abuse, security, maladaptive cognitions, and pathological Internet use are presented in Table 1, which also presents the associations between these factors. Child abuse was negatively associated with security and positively associated with maladaptive cognitions and pathological Internet use. Security was negatively associated with maladaptive cognitions and pathological Internet use. Maladaptive cognitions and pathological Internet use were positively associated with each other. Additionally, childhood abuse score was rage from 28 to 97; security score was rage from 16 to 66; maladaptive cognitions score was rage from 14 to 70; pathological Internet use score was rage from 19 to 76.

**TABLE 1:** Descriptive statistics and correlation analysis of variables.

Variables	1	2	3	4
Child abuse	1			
Security	−0.35**	1		
Maladaptive cognitions	0.27**	−0.49**	1	
Pathological Internet use	0.35**	−0.49**	0.68**	1
M	41.43	34.55	32.02	32.95
SD	10.04	10.61	9.96	10.52

**p < 0.01.

Gender differences were tested in studied variables to identify whether gender should be regarded as controlled variable during the later analysis. Gender differences in child abuse, security, maladaptive cognitions, and pathological Internet use were examined with a series of independent-samples *t*-tests; male students experienced higher levels of child abuse than did female students, and no gender differences were observed for security, maladaptive cognitions, or pathological Internet use (Table 2).

**TABLE 2:** Gender difference test of variables.

Variables	Male (*M* ± *SD*)	Female (*M* ± *SD*)	*t*	*df*	*P*	Cohen’s *d*
Child abuse	43.13 ± 11.86	40.15 ± 8.18	4.50	916	<0.01	0.29
Security	34.26 ± 11.13	34.76 ± 10.21	–0.70	916	0.48	–0.05
Maladaptive cognitions	31.86 ± 10.43	32.14 ± 9.60	–0.42	916	0.68	–0.03
Pathological Internet use	33.03 ± 11.09	32.90 ± 10.08	0.19	916	0.85	0.01

### Mediating Roles of Security and Maladaptive Cognitions

**FIGURE 2:**
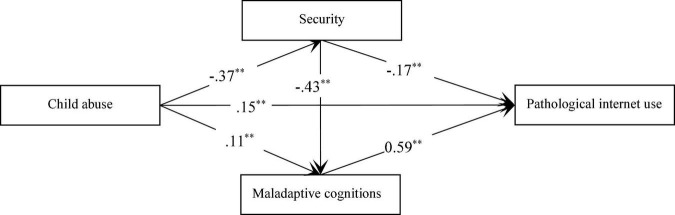
Confirmed model. ^∗∗^*p* < 0.01.

The mediating roles of security and maladaptive cognitions in the association between child abuse and pathological Internet use were tested using SEM (Figure 2). According to previous study’s suggestion (Zeng et al., 2021), gender was coded as controlled variable was included into the SEM. The results of which demonstrated the model achieved favorable fit indices (χ^2^[3] = 2.12, *p* < 0.001, RMSEA = 0.035, TLI = 0.99, CFI = 0.99). Additionally, the model indicated that child abuse could positively predict pathological Internet use (β = 0.15, SE = 0.03, *p* < 0.01) and maladaptive cognitions (β = 0.11, SE = 0.04, *p* < 0.01) and negatively predict security (β = −0.35, SE = 0.03, *p* < 0.01). Security could negatively predict pathological Internet use (β = −0.17, SE = 0.03, *p* < 0.01) and maladaptive cognitions (β = −0.45, SE = 0.03, *p* < 0.01), and maladaptive cognitions could positively predict pathological Internet use (β = 0.56, SE = 0.03, *p* < 0.01). To examine the mediating roles of security and maladaptive cognitions in the association between child abuse and pathological Internet use, a bootstrapping test was conducted. Studies have suggested a significant mediating role at the 0.05 level if the 95% confidence level does not include zero (Shrout and Bolger, 2002). The results revealed that child abuse could positively predict pathological Internet use through the mediating roles of security (95% confidence interval [CI]: 0.04–0.08), maladaptive cognitions (95% CI: 0.03–0.10), and the chain mediating role of security and maladaptive cognitions (95% CI: 0.08–0.12).

## Discussion

### Association Between Child Abuse and Pathological Internet Use

This study revealed that child abuse could positively predict pathological Internet use, which, to some extent, is consistent with the results of previous studies (Siomos et al., 2012; Chen et al., 2017; Lo et al., 2021). According to the social compensation model, pathological Internet use could compensate for a lack of personal relationships, particularly parent–child relationships, which could lead to insecure attachment, emotional disorders, and cognitive dysfunction (Morgan and Cotton, 2004; Frison and Eggermont, 2016). The social and entertainment functions of the Internet could rectify deficiencies in interpersonal relationships experienced during childhood. For example, computer games could relieve negative emotions, and positive emotional experiences could help individuals to forget poor childhood experiences. Although individuals who experienced child abuse also experienced more positive than negative emotions, they tended to spend more time on the Internet, which led to subsequent pathological Internet use (Siomos et al., 2012; Chen et al., 2017; Lo et al., 2021). Although studies have examined the association between child abuse and pathological Internet use, they have tended to focus on children (Siomos et al., 2012) or adolescents (Lo et al., 2021). The present study, however, sampled university students, with the results revealing that pathological Internet seems to be use in order to try to compensate for childhood abuse.

### Mediating Roles of Security and Maladaptive Cognitions

The current results indicated the new notion that child abuse could positively predict individuals’ pathological Internet use through the mediating role of security. Studies have suggested that the Internet meets people’s social communication function to a great extent. A rich and convenient lifestyle can reduce uncertainty in people’s lives and effectively meet their sense of security in both interpersonal communications and life in general (Hadlington and Parsons, 2017; Jia et al., 2017). Although child abuse reportedly had a significant negative association with individuals’ senses of security (Boisjoli et al., 2019; Tierolf et al., 2020), those who experienced child abuse tended to use the Internet more often to search for security. Moreover, the present study indicated that child abuse could positively predict individuals’ pathological Internet use through the mediating role of maladaptive cognitions. According to the cognitive behavioral model, maladaptive cognitions are the proximal sufficient cause of pathological Internet use. Individuals who experienced child abuse tend to develop pathological Internet use through increasing maladaptive cognitions regarding the Internet (Davis, 2001; Caplan, 2002). Those who experienced abuse may be more likely to develop emotional disorders, and the entertainment function of the Internet then helps relieve these negative emotions. As these individuals’ negative emotions are relieved through Internet use, they become susceptible to developing maladaptive cognitions regarding the Internet, such as “the Internet can make my life more comfortable than the real world.” These cognitions may lead these individuals to use the Internet more often than other people do.

Furthermore, this study determined that child abuse could positively predict pathological Internet use through the chain mediating role of security and maladaptive cognitions. The results indicated that individuals with a low sense of security tended to develop pathological Internet use following an increase in their maladaptive cognitions about the Internet. A rich and convenient lifestyle may reduce uncertainty in people’s lives and effectively increase their sense of security about interpersonal communication and life (Hadlington and Parsons, 2017; Jia et al., 2017). Although individuals who experienced child abuse obtained an increased sense of security through their use of the Internet, they tended to develop more maladaptive cognitions regarding the Internet, such as “the Internet is safer than reality,” than other people, which may lead them to develop pathological Internet use. The present results also contribute to the development of the cognitive behavioral model because child abuse and security tend to be two essential distal causes, rather than proximal sufficient causes, of pathological Internet use.

### Implications for School Achievement

Previous study have found problematic Internet use had negative influences on students’ school achievement (Davis, 2001; Caplan, 2002), and according to the present findings, two implications for the treatment and prevention of problematic Internet use to improve university students’ school achievement are provided. First, the school or family should help university student with child abuse experience build a strong sense of security to overcome the negative influences of problematic Internet use on students’ school achievement. Second, the school or family should help university student with child abuse experience decrease the maladaptive cognitions to overcome the negative influences of problematic Internet use on students’ school achievement.

### Contributions, Limitations, and Future Directions

There are two important contributions of this study. First, although previous studies have indicated that child abuse is associated with addictive behaviors (Siomos et al., 2012; Chen et al., 2017; Lo et al., 2021), no study have tested the effect of child abuse on pathological Internet use and the results of present study have filled this gap. Second, the results of present study have found child abuse could predict pathological Internet use through the sequential mediating roles of security and maladaptive cognitions, which indicated both child abuse and security tended to be important distal predictors of pathological Internet use in cognitive behavioral model, which further expanded the model. This study also has several limitations. First, because a cross-sectional design was employed, no causal inferences were determined. Future studies could use experimental designs to further verify the results of the present study. Second, all participants were from one university, which may limit the generalizability of the results; therefore, future studies could use other samples in China or other countries to verify the current results. Third, previous studies have suggested that university students are more likely than other individuals to exhibit pathological Internet use because have they have more free time (Morahan-Martin and Schumacher, 2000; Chen, 2012), while the present study have not measured the free time of all the participants. Therefore, future study could test the influence of individuals’ free time on pathological Internet use.

## Conclusion

This study can draw two conclusions. First, child abuse is an important predictor of pathological Internet use. Second, security and maladaptive cognitions are two important mediating factors in the association between child abuse and pathological Internet use.

## Data Availability Statement

The original contributions presented in the study are included in the article/supplementary material, further inquiries can be directed to the corresponding authors.

## Ethics Statement

The present study was conducted in accordance with the 1964 Helsinki declaration and its later amendments or comparable ethical standards, with the approval of the Human Research Ethics Committee of Qingdao University of Science and Technology. The patients/participants provided their written informed consent to participate in this study.

## Author Contributions

NQ collected the data and wrote and polished the manuscript. PL polished the manuscript. YT designed the present study and wrote and polished manuscript. All authors contributed to the article and approved the submitted version.

## Conflict of Interest

PL was employed by company of Tsingtao Beer Museum. The remaining authors declare that the research was conducted in the absence of any commercial or financial relationships that could be construed as a potential conflict of interest.

## Publisher’s Note

All claims expressed in this article are solely those of the authors and do not necessarily represent those of their affiliated organizations, or those of the publisher, the editors and the reviewers. Any product that may be evaluated in this article, or claim that may be made by its manufacturer, is not guaranteed or endorsed by the publisher.
